# Interleukin-18 and High-Mobility-Group-Protein B1 are Early and Sensitive Indicators for Cell Damage During Normothermic Machine Perfusion after Prolonged Cold Ischemic Storage of Porcine Liver Grafts

**DOI:** 10.3389/ti.2022.10712

**Published:** 2022-10-20

**Authors:** Oliver Beetz, Sebastian Cammann, Clara A. Weigle, Lion Sieg, Hendrik Eismann, Kai Johanning, Christine S. Falk, Till Krech, Felix Oldhafer, Florian W. R. Vondran

**Affiliations:** ^1^ Department of General, Visceral and Transplant Surgery, Hannover Medical School, Hannover, Germany; ^2^ Department of Anesthesiology and Intensive Care Medicine, Hannover Medical School, Hannover, Germany; ^3^ Institute of Transplant Immunology, Hannover Medical School, Hannover, Germany; ^4^ German Center for Infection Research, DZIF, TTU-IICH Braunschweig Site, Hannover, Germany; ^5^ German Center for Lung Research DZL, BREATH, Hannover, Germany; ^6^ Department of Pathology, University Medical Centre Hamburg-Eppendorf, Hamburg, Germany

**Keywords:** ischemia-reperfusion injury, normothermic machine perfusion, cytokine, extended criteria donor organs, marginal organs, IL-18, HMGB1

## Abstract

In the era of organ machine perfusion, experimental models to optimize reconditioning of (marginal) liver grafts are needed. Although the relevance of cytokine signatures in liver transplantation has been analyzed previously, the significance of molecular monitoring during normothermic machine perfusion (NMP) remains elusive. Therefore, we developed a porcine model of cold ischemic liver graft injury after prolonged static cold storage (SCS) and subsequent NMP: Livers obtained from ten minipigs underwent NMP for 6 h directly after procurement (control group) or after 20 h of SCS. Grafts after prolonged SCS showed significantly elevated AST, ALT, GLDH and GGT perfusate concentrations, and reduced lactate clearance. Bile analyses revealed reduced bile production, reduced bicarbonate and elevated glucose concentrations after prolonged SCS. Cytokine analyses of graft perfusate simultaneously demonstrated an increase of pro-inflammatory cytokines such as Interleukin-1α, Interleukin-2, and particularly Interleukin-18. The latter was the only significantly elevated cytokine compared to controls, peaking as early as 2 h after reperfusion (11,012 ng/ml vs. 1,493 ng/ml; *p* = 0.029). Also, concentrations of High-Mobility-Group-Protein B1 were significantly elevated after 2 h of reperfusion (706.00 ng/ml vs. 148.20 ng/ml; *p* < 0.001) and showed positive correlations with AST (*r* ^2^ = 0.846) and GLDH (*r* ^2^ = 0.918) levels. Molecular analyses during reconditioning of liver grafts provide insights into the degree of inflammation and cell damage and could thereby facilitate future interventions during NMP reducing acute and chronic graft injury.

## Graphical Abstract

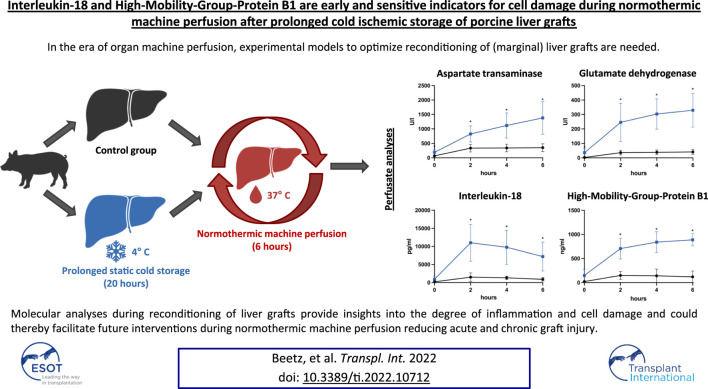

## Introduction

In an era of worldwide organ shortage solutions to expand the donor pool and to reduce waitlist mortality of patients with end-stage liver disease are desperately needed. Accordingly, the increased utilization of marginal or extended criteria donor (ECD) organs has become clinical reality in many countries. Although there is no universally accepted definition of such organs, different donor-, graft- or storage-associated factors indicating a suboptimal graft quality, such as advanced donor age, hepatic steatosis or prolonged static cold storage (SCS) prior transplantation are most commonly being taken into account [1]. The short- and long-term success of this approach is critically limited by a higher vulnerability of ECD organs to an inevitable ischemia-reperfusion injury (IRI) induced and aggravated by the current state-of-the-art SCS and related early allograft dysfunction, bile duct complications and chronic allograft dysfunction [2, 3]. Hence, new challenges regarding optimal organ preservation and reconditioning have emerged.

The rediscovery and technical evolution of machine perfusion has the potential not only to adequately meet these demands but also to revolutionize the fields of organ repair, modification and, ultimately, transplantation.

Various forms of machine perfusion have been introduced and evaluated. Most prominently, the hypothermic (oxygenated) and the normothermic machine perfusion (NMP) are on the verge of entering clinical routine after being successfully assessed in large prospective multicenter trials [4, 5]. Although hypothermic machine perfusion, applying 4°C cold preservative (oxygenated) solution, has proven to be feasible and safe in several trials and reduces biliary complications as well as early allograft dysfunction, temperature-dependent downregulation of cellular metabolism impedes testing of hepatic function during perfusion, so far. NMP, on the other hand, is a more demanding technical procedure as it usually requires blood-based oxygenated perfusates at 37°C but thereby allows physiological aerobic metabolism and, hence, viability assessment of grafts prior to transplantation, which is essential to determine whether an ECD organ can be utilized or has to be discarded. Conventional parameters such as concentrations of aspartate or alanine transaminase and lactate, pH, glucose metabolism, bile production, adequate flow rates and a homogenous perfusion are currently applied experimentally and clinically, for example within the prospective VITTAL study [6, 7]. Although the relevance of cytokine signatures and damage-associated molecular patterns (DAMPs) in liver transplantation has been analyzed in the past [8, 9], the role of molecular monitoring during NMP and immunomodulatory effects of NMP especially on ECD organs remains surprisingly elusive. Ferdinand et al. recently reported increased inflammatory gene expression during NMP of human kidney grafts and demonstrated improved graft function upon adsorption of pro-inflammatory mediators [10]. In order to study these mechanisms and to evaluate therapeutic options for (marginal/ECD) liver grafts in the future, we developed a porcine model of cold ischemic liver graft injury after prolonged SCS of 20 h and subsequent NMP and investigated the inflammatory milieu in the perfusates.

## Materials and Methods

### Legal Approval

This study was performed at the Laboratory for Animal Science of Hannover Medical School after approval by the Lower Saxony regional authority for consumer protection and food safety (Niedersächsisches Landesamt für Verbraucherschutz und Lebensmittelsicherheit (LAVES); 19/3146). The animals were kept under housing conditions of the EU-Guideline 2010/63 and valid German animal regulation act (Tierschutz-Versuchstierverordnung des deutschen Tierschutzgesetzes).

### Experimental Design

The primary objective of this study was to introduce an experimental porcine model of cold ischemic liver graft injury after prolonged SCS in order to characterize the release of pro- and anti-inflammatory molecules during (re)perfusion under NMP.

**FIGURE 1:**
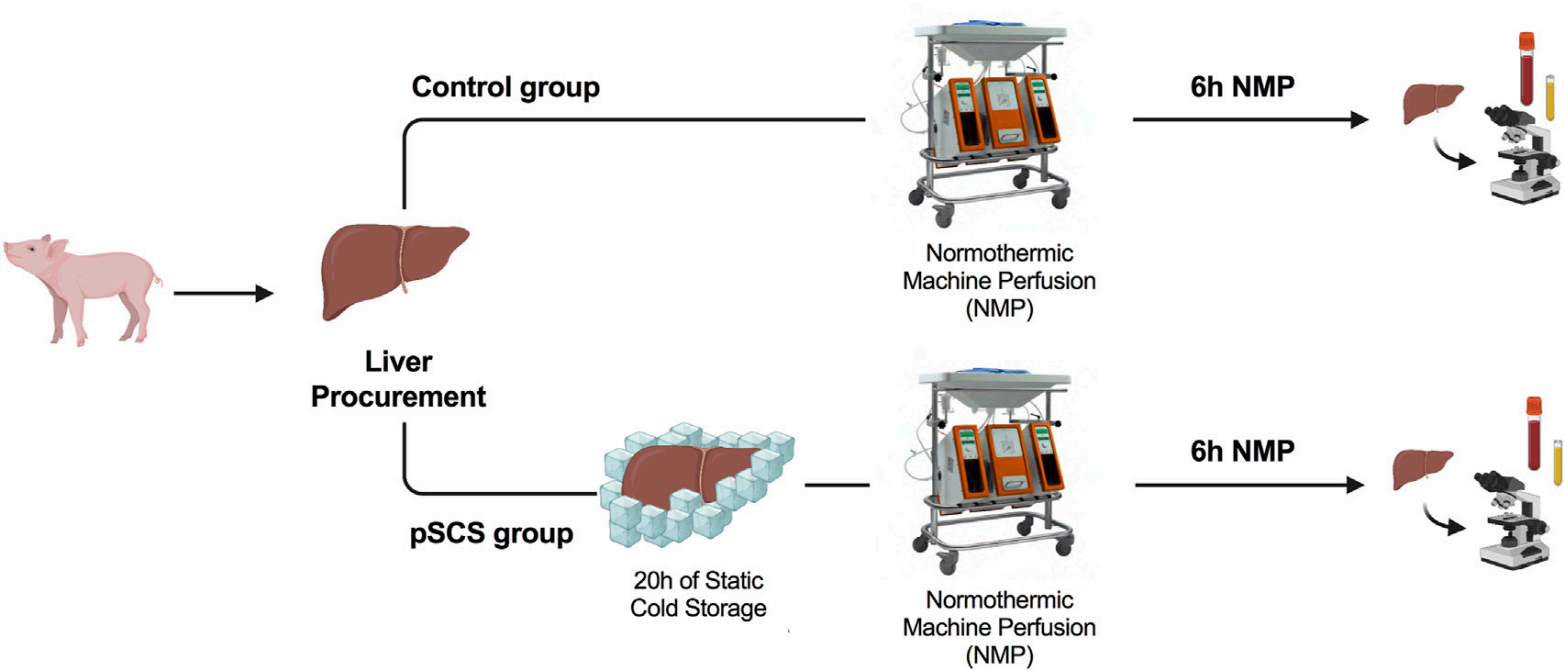
Study design and time points of interventions. Normothermic machine perfusion (NMP) was performed directly (control group) or 20 h after liver procurement and static cold storage (prolonged SCS (pSCS) group), respectively. After 6 hours, animals were euthanized, serum samples were taken and liver and bile duct tissue was recovered for histological work-up.

Healthy pigs were divided into two groups: In the control group NMP was performed directly after liver procurement. In the group of prolonged SCS NMP was initiated after 20 h of SCS (4°C). All livers were perfused for a total of 6 hours. The experimental design is displayed in Figure 1.

### Liver Procurement and Back-Table Preparation

Healthy female LEWE minipigs (*n* = 10) with a median age of 118 (117–126) days and median body weight of 52 (49–57) kg were used as liver donors. After premedication with zolazepam (5 mg/kg bodyweight) and atropine (0.02–0.04 mg/kg bodyweight) *via* intramuscular injection, anesthesia was induced by intravenously applied propofol (1.5–2.5 mg/kg bodyweight). Upon endotracheal intubation anesthesia and analgesia were maintained with isoflurane (0.8–1.5 vol%) and fentanyl (0.003–0.007 mg/kg bodyweight) as previously reported [11, 12]. The procurement of the liver was performed as described in humans [13]. In brief, first the abdominal aorta and the inferior vena cava were exposed and the perfusion cannula was inserted cranial of the aortic bifurcation. Thereafter, the left lateral liver lobe was mobilized and upon transverse incision of the diaphragm the thoracic aorta was encircled. Before cross clamping of the supra-coeliac aorta, 25,000 I.E. heparin were administered intravenously. Exsanguination of the donor was then achieved by dissection of the suprahepatic vena cava inferior and collection of approximately 1,500 ml of blood in a container containing citrate-based anticoagulant (citrate-phosphat-dextrose solution with adenine). Afterwards, cold antegrade perfusion was performed with 3,500 to 4,000 ml of Custodiol (HTK)-solution (Dr. Franz Köhler Chemie GmbH, Bensheim, Germany) over a course of approximately 10–15 min followed by retrieval of the liver. Animals additionally received an intravenous bolus injection of a lethal dose (5000 mg) of pentobarbital sodium for intraoperative euthanasia.

**FIGURE 2:**
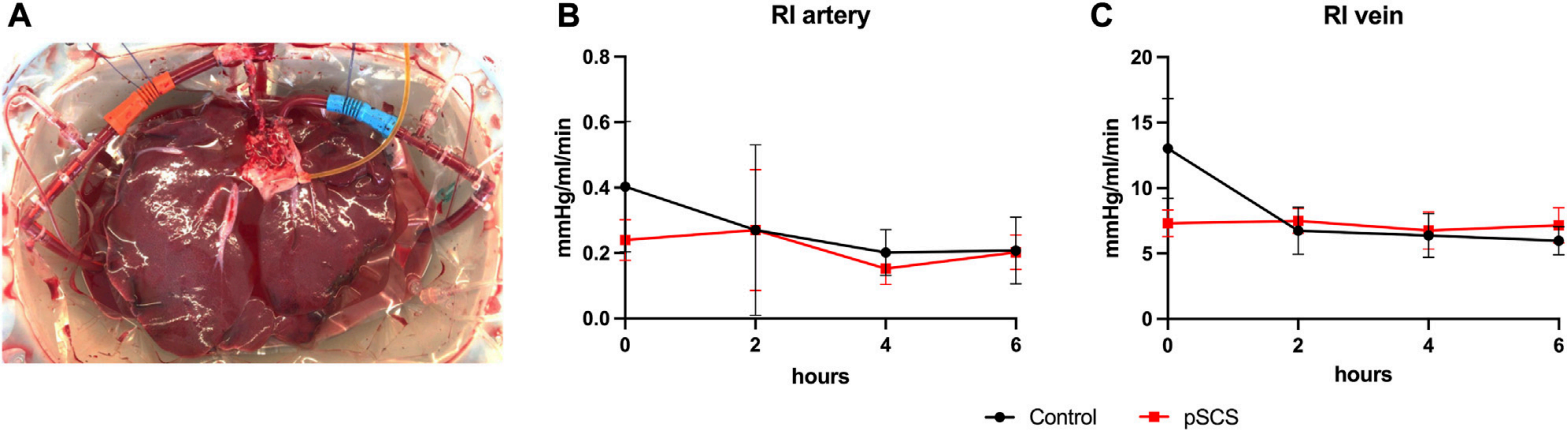
Experimental set-up of the NMP. A representative example of a homogenous perfusion of a liver graft over the duration of 6 hours is depicted **(A)**. Courses of the computed arterial **(B)** and venous **(C)** resistance index (RI) during 6 hours of NMP are shown. Comparison of control group (black line) with prolonged SCS (pSCS) group (red line).

After organ retrieval, the aortic segment was closed to one side by a doubled running non-absorbable monofilament suture (4-0 Prolene) and the aortic cannula was inserted on the opposite side and secured with a single purse-string suture (4-0 Prolene). Side branches of the aortic segment, the coeliac trunk and the hepatic artery were occluded with titanium clips. The portal vein cannula was inserted and secured in a similar fashion (Figure 2A). After ligation of the cystic duct, the common bile duct was cannulated and flushed with at least 20 ml of cold Custodiol (HTK)-solution.

### Normothermic Machine Perfusion

NMP was performed with a Liver Assist device (Organ Assist, Groningen, the Netherlands). Approximately 1,500 ml of autologous whole blood, collected as described above, was used for perfusion. Colloid solution (e.g., Gelafundin) was added to achieve a total volume of approximately 2,000 ml. The perfusate was set to a temperature of 37°C and oxygenated with 100% oxygen at 0.5–1.0 L/min gas flow. The portal vein pressure was set at 8 mmHg. The hepatic artery was perfused with a pulsatile flow at a pressure of 60 mmHg. Potassium, insulin, calcium gluconate and sodium bicarbonate were added during the perfusion in order to achieve physiological conditions. Bile production was measured every 60 min and samples were taken every 2 hours after reperfusion (0, 2, 4, and 6 h).

### Conventional Laboratory Parameters

Blood gas analyses (including lactate concentrations) were performed before and every 30 min after reperfusion. Aspartate transaminase (AST), Alanine transaminase (ALT), Glutamate dehydrogenase (GLDH), Alkaline phosphatase (ALP), Gamma-glutamyltransferase (GGT), bilirubin, urea and creatinine serum concentrations of perfusate samples were analyzed before and every 2 hours after the begin of perfusion.

### Histological Analyses

Tissue samples for histology were obtained from the liver and the bile duct prior to reperfusion and after 6 hours of reperfusion. The tissue was fixed in buffered 4% formaldehyde and subsequently embedded in paraffin according to standard histopathological protocols. For histologic evaluation 4 µm thick sections were cut and stained with hematoxylin and eosin. Liver sections were semiquantitatively analyzed for the degree of inflammation (absent, mild, moderate, severe) as described by Ali et al [14]. Bile duct injury was assessed using a scoring system described by Hansen et al. and modified by op den Dries et al. ([15, 16]; see also Table 1). The histological samples were evaluated by a liver pathologist (TK) using a Zeiss Axio Imager A2 microscope, field number 25 (Zeiss, Germany). TK was blinded to the operative procedures. Microphotographs were generated using a Hamamatsu Nano zoomer S360 digital slide scanner (Hamamatsu, Japan).

**TABLE 1:** Applied scoring system for bile duct injury.

Bile duct wall component	Grade 0	Grade 1	Grade 2	Grade 3
Biliary epithelium	No loss	≤50% loss	>50% loss	n.a.
Mural stroma	No injury	≤25% necrotic	25–50% necrotic	>50% necrotic
Peribiliary vascular plexus	No injury	≤50% of vessels with changes	>50% of vessels with changes	Grade 2 + arteriolonecrosis
Thrombosis	Absent	Present	n.a.	n.a.
Intramural bleeding	None	≤50% of duct wall	>50% of duct wall	n.a.
Periluminal PBG	No injury	≤50% loss of cells	>50% loss of cells	n.a.
Deep PBG	No injury	≤50% loss of cells	>50% loss of cells	n.a.
Inflammation	None	At least 10 leukocytes/HPF	At least 50 leukocytes/HPF	n.a.

PBG, peribiliary glands; HPF, high-power field.

### Cytokine Multiplex Analyses

Luminex-based multiplex technology (Milliplex Porcine Cytokine/Chemokine Premixed 13-Plex Magnetic Bead Kit, Merck, United States) was used to generate cytokine profiles of perfusates, as previously reported [17]. Bio-Plex Manager 6.0 software was used to calculate standard curves and cytokine concentrations. The detection limit of all proteins was 1–10 pg/ml.

### Enzyme-Linked Immunofluorescent Assays

High-Mobility-Group-Protein B1 (HMGB1) serum concentrations were measured before and every 2 hours after reperfusion using a commercially available ELISA kit (Reference Number ST51011, Tecan—IBL International, Hamburg, Germany).

### Statistical Analyses

Statistical analysis was performed using GraphPad PRISM 8.4.00 (GraphPad Software, Inc., La Jolla, CA). Comparison of mean values between both groups were performed with the Student’s *t*-test in case of normal distribution or the Mann-Whitney *U* test, respectively. Differences were regarded statistically significant at p-values of < 0.050. Correlation between variables were expressed by the Pearson correlation coefficient. Results are expressed as mean ± standard deviation (SD) unless indicated otherwise.

## Results

### Perfusion Parameters Were Not Influenced by Prolonged Static Cold Storage

None of the perfusions had to be terminated prematurely due to technical difficulties. Liver grafts of both groups showed homogenous perfusion over the duration of 6 h (Figure 2A).

Accordingly, computed resistance index (RI) did not vary over time and did not differ significantly between the prolonged SCS and the control group (Figures 2B,C). The mean arterial RI was 0.208 mmHg/L/min in the control group and 0.203 mmHg/L/min in the prolonged SCS group (*p* = 0.926). After 6 h of perfusion, the mean venous RI was 5.972 mmHg/L/min in the control group and 7.155 mmHg/L/min in the prolonged SCS group (*p* = 0.184).

### Conventional Liver Function Parameters Were Elevated by Prolonged Static Cold Storage

**FIGURE 3:**
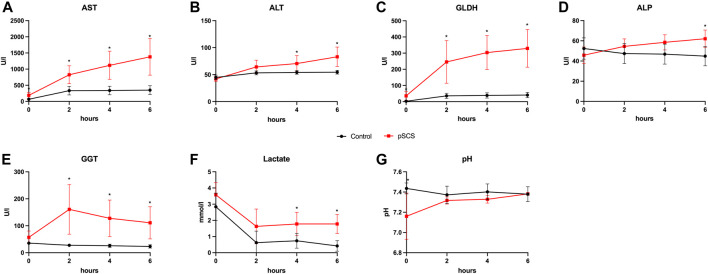
Courses of perfusate parameters during NMP. **(A–G)** Courses of AST, ALT, GLDH, ALP, GGT, lactate and pH during 6 hours of perfusion are shown. Comparison of control group (black line) with prolonged SCS (pSCS) group (red line). *: *p* < 0.050.

Perfusate samples of grafts undergoing perfusion after SCS for 20 h revealed higher concentrations of AST, ALT and GLDH with an increasing difference over time, when compared to control grafts (control group): after 6 h of perfusion, the mean perfusate concentrations of AST, ALT and GLDH in the control group were 350.30 U/l, 54.50 U/l and 41.17 U/l, respectively, and stable throughout the previous 4 h of perfusion, whereas mean perfusate concentrations in the prolonged SCS group were 1,380.00 U/l, 83.00 U/l and 329.30 U/l (*p* = 0.010; *p* = 0.024; *p* = 0.024), respectively, and were rising throughout the length of perfusion (Figures 3A–C).

Accordingly, ALP and GGT perfusate concentrations indicating biliary damage were also elevated after prolonged SCS (Figures 3D,E). In this context, it is particularly noteworthy that the concentrations of GGT reached a significantly higher level as early as 2 h after reperfusion when comparing both groups (160.80 U/l vs 27.67 U/l; *p* = 0.010).

Although lactate concentrations declined after 2 h and remained stable under 2 mmol/L with ongoing perfusion in both groups, grafts with prolonged SCS showed significantly reduced lactate clearance after 6 h of perfusion (1.775 mmol/L vs 0.417 mmol/L, *p* = 0.010; Figure 3F). The perfusate pH was kept stable throughout the perfusion in both groups (Figure 3G).

### Bile Composition and Production Were Impaired by Prolonged Static Cold Storage

**FIGURE 4:**
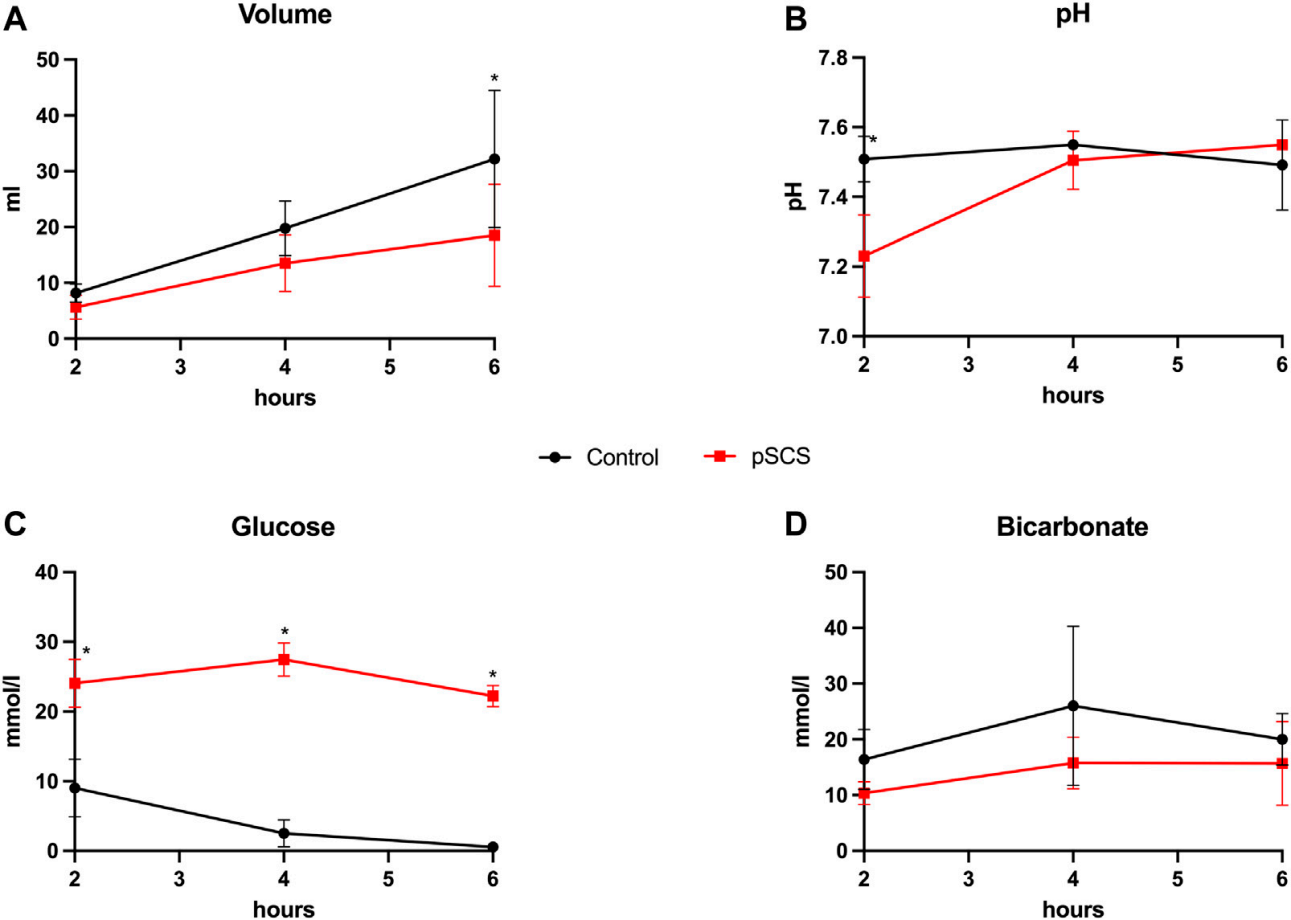
Bile production and composition during NMP. **(A–D)** Courses of bile volume, pH, glucose and bicarbonate during 6 hours of perfusion are shown. Comparison of control group (black line) with prolonged SCS (pSCS) group (red line). *: *p* < 0.050.

In line with the biliary damage indicated by the above mentioned perfusate analyses, grafts after prolonged SCS exhibited reduced bile production of only 18.50 ml compared to 32.20 ml (*p* = 0.067) over the course of perfusion, whereas analyses of the bile composition revealed lower bicarbonate concentrations by trend (19 mmol/L vs. 22 mmol/L; *p* = 0.625) and significantly higher glucose concentrations (22.20 mmol/L vs. 1.30 mmol/L; *p* = 0.024; Figure 4).

### Pro-Inflammatory Cytokines and HMGB-1 Were Increased After Prolonged Static Cold Storage

**FIGURE 5:**
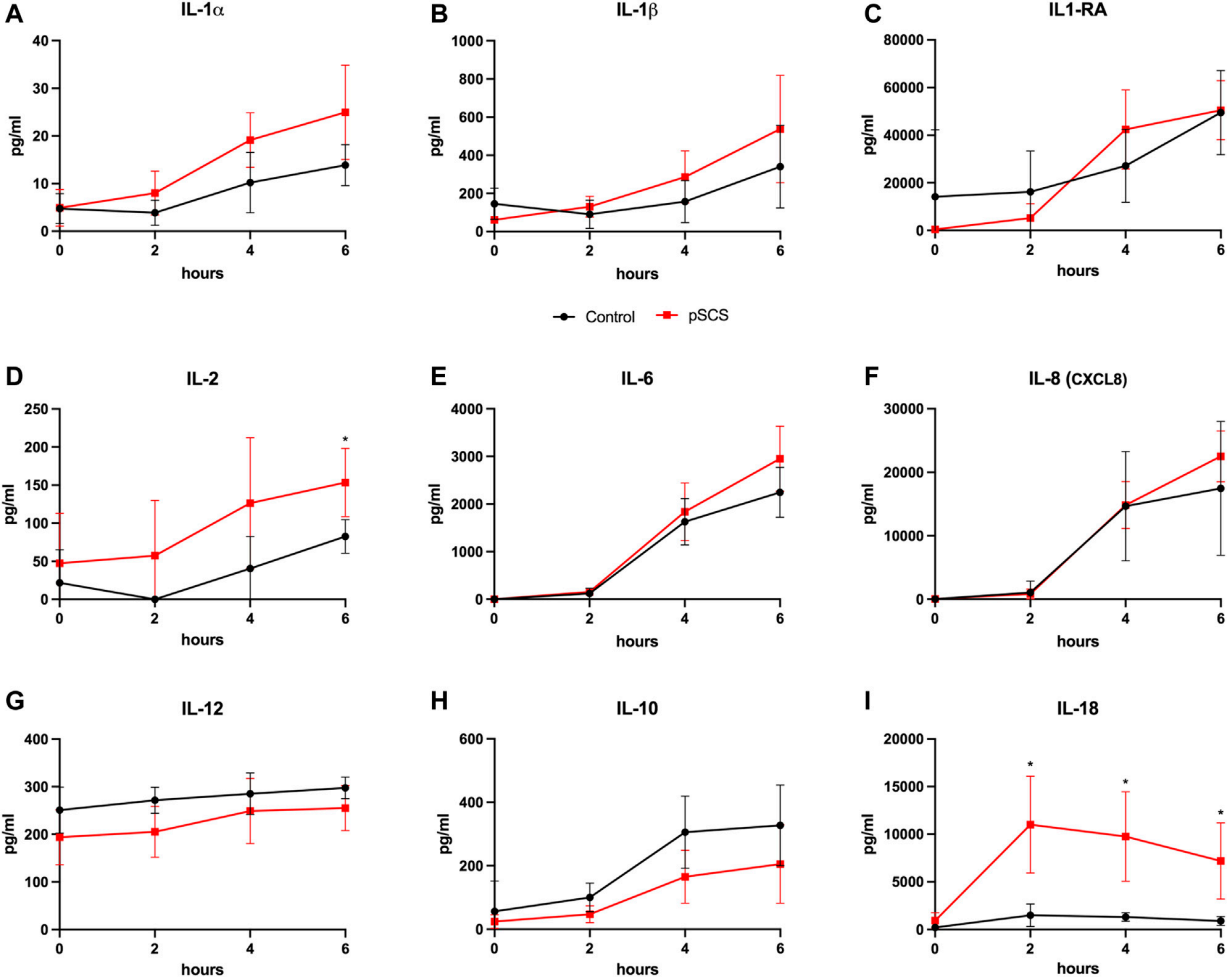
Courses of cytokines during NMP. **(A–I)** Courses of IL-1α, IL-1β, IL-2, IL-6, IL-8, IL-12, IL-10 and IL-18 during 6 hours of perfusion are shown, respectively. Comparison of the control group (black line) with the prolonged SCS (pSCS) group (red line). *: *p* < 0.050.

A comparison of cytokine perfusate concentrations between both groups showed higher values but no significant differences regarding the pro-inflammatory cytokines IL-1α, IL-1β, IL-2, IL-6 and the chemokine CXCL8 (IL-8) at the end of perfusion in grafts with prolonged SCS (Figures 5A–F). IL-12 was the only pro-inflammatory cytokine with numerically higher values in the control group compared to the prolonged SCS group without significant differences after 6 h (297.5 pg/ml vs 255.3 pg/ml, *p* = 0.200) (Figure 5G). On the contrary, concentrations of the anti-inflammatory cytokine IL-10 were continuously lower in the prolonged SCS group, without reaching statistical significance (Figure 5H). More pronounced and significant differences, respectively, were observed for IL-18 and HMGB1 perfusate concentrations: The caspase-cleavage-dependent pro-inflammatory cytokine IL-18 increased after reperfusion, with an early peak after 2 h with 11,012.0 pg/ml and a slow decrease thereafter (7,195.0 pg/ml after 6 h) in the prolonged SCS group compared to stable concentrations of 1,493.0 pg/ml after 2 h (*p* = 0.029) and 896.5 pg/ml after 6 h (*p* = 0.029) in the control group (Figure 5I).

**FIGURE 6:**
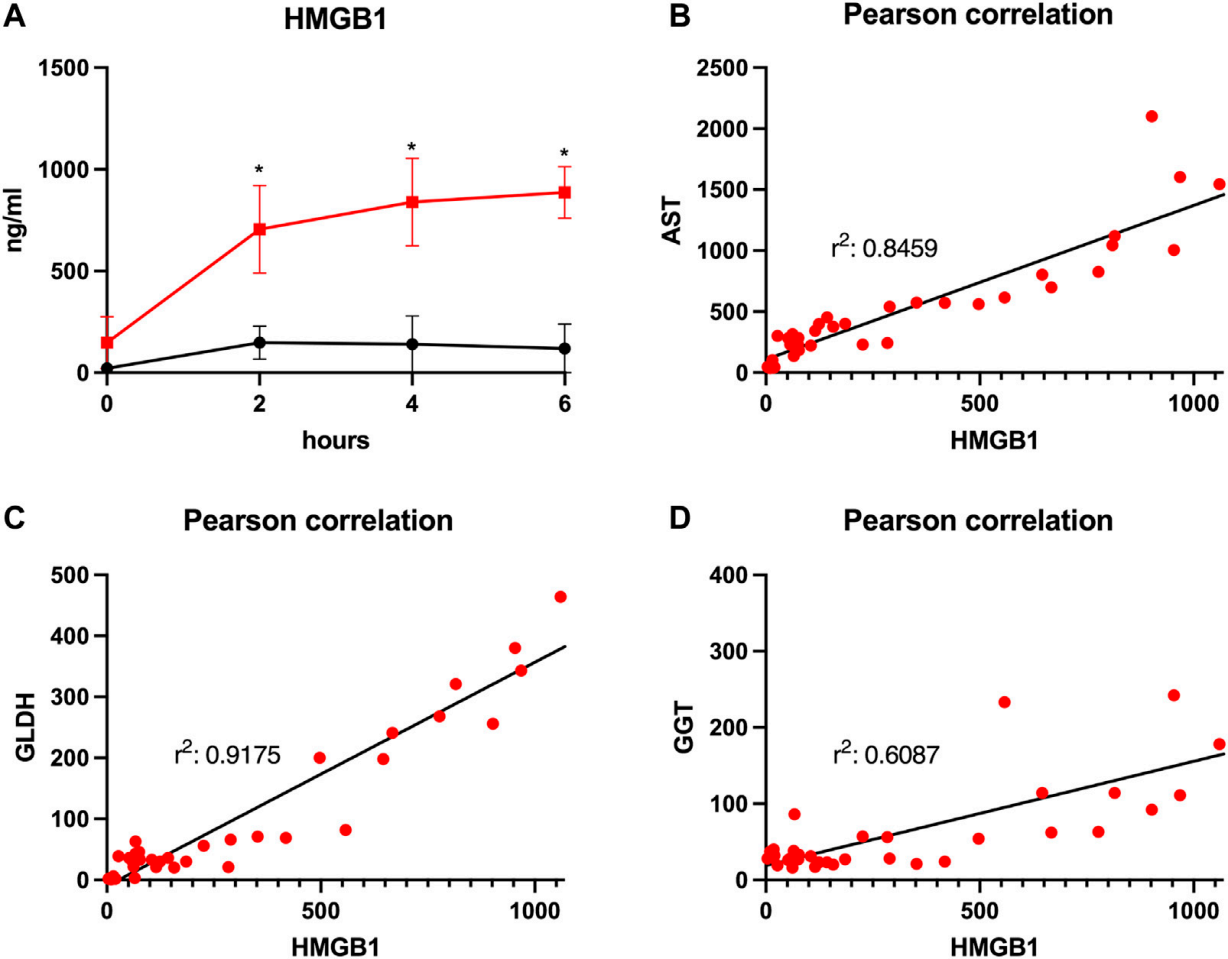
Courses of HMGB1 during 6 h of perfusion are shown **(A)**. Comparison of the control group (black line) with the prolonged SCS (pSCS) group (red line). *: *p* < 0.050. Pearson correlations for HMGB1 with AST **(B)**, GLDH **(C)** and GGT **(D)**, respectively, are shown.

HMGB1, indicating inflammation and tissue damage, also rapidly increased during NMP after prolonged SCS and reached a plateau towards the end of perfusion with 887.3 pg/ml after 6 h (Figure 6A). HMGB1 perfusate concentrations in the control group peaked and then declined after approximately 2 h reaching a mean concentration of 119.3 pg/ml at the end of perfusion (*p* = 0.010). Of note, the increase in HMGB1 perfusate concentrations correlated well with liver function parameters such as AST and GLDH indicating hepatocyte and endothelial damage (*r* ^2^ = 0.846 and *r* ^2^ = 0.918, respectively; Figures 6B,C) as well as with GGT concentrations, indicating biliary injury (*r* ^2^ = 0.609; Figure 6D).

The following cytokines were detected with very low values in the majority of the obtained perfusate samples and were therefore not further analyzed: TNF-α, IL-4 and IFN-γ.

### Histological Analyses

**FIGURE 7:**
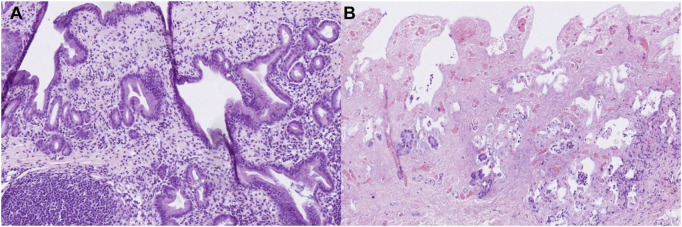
Representative histological sections (hematoxylin-eosin staining, original magnification ×200) of bile ducts obtained from grafts undergoing 6 hours of NMP immediately after procurement **(A)** or after 20 h of SCS **(B)**. **(A)** shows a vital bile duct with dense inflammatory infiltrates (grade 2). **(B)** shows a largely necrotic bile duct without surface epithelium (grade 3), extensive necrosis of the mural stroma (grad 3), extensive damage to the periluminal peribiliary glands (grade 3) and mild inflammatory infiltrates (grade 1).

Histological analyses of liver and bile duct specimens obtained before and after 6 h of NMP did not reveal statistically significant differences between both groups, most likely as a result of the semiquantitative histological scoring system applied (see Table 1), the comparatively small sample number and statistical outliers for each histological item. However, there was an increase in the degree of liver inflammation by trend, which was largely absent prior to perfusion and increased to a mild or moderate degree in both groups, respectively. Furthermore, grafts after prolonged SCS showed increased biliary damage after 6 h of NMP when compared to control grafts. In more detail, there was a trend towards a higher degree of injury concerning the biliary epithelium (median (range): 2 (2) vs. 1 (1–2); *p* = 0.200), mural stroma (median (range): 2 (1–3) vs. 1 (0–2); *p* = 0.500) and periluminal peribiliary glands (median (range): 2 (1–2) vs. 1 (0–2); *p* = 0.700). Figures 7A,B depicts representative histological sections obtained from bile ducts of both groups after 6 h of NMP illustrating the spectrum of biliary damage.

## Discussion

Cold and warm ischemia during organ retrieval, storage and reperfusion inevitably results in IRI of liver grafts, i.e., hepatocytes, cholangiocytes, liver sinusoidal endothelial cells and non-parenchymal resident immune cells. The subsequent release of pro-inflammatory cytokines, such as IL-1β, IL-6, and CXCL8/IL-8 as well as DAMPs and reactive oxygen species initiates the development of an inflammatory environment: neutrophil recruitment and activation of Kupffer cells, leading to a further secretion of IL-1β and TNF-α and upregulation of adhesion molecules such as Mac-1 and ICAM-1, further promotes the infiltration of immune cells into the liver parenchyma and induces additional parenchymal injury [18, 19].

The current gold standard of organ preservation represented by SCS and an ongoing organ donor shortage with increased use of ECD organs induce or aggravate these mechanisms. In contrast, a variety of experimental and clinical studies have demonstrated that machine perfusion ameliorates the detrimental effects of IRI on liver grafts with regard to early allograft dysfunction or ischemic biliary complications [20, 21]. Most recently, Markmann et al. published data of a randomized clinical multicenter trial, including 293 patients, displaying a significant reduction of lobular inflammation after graft reperfusion with previous NMP [22]. Accordingly, Jassem et al. observed reduced numbers of pro-inflammatory cytokine producing T-cells among donor lymphocytes and higher numbers of CD4^pos^CD25^high^CD127^neg^FOXP3^pos^ regulatory T-cells in the perfusate of 12 liver grafts after NMP, when compared to 27 grafts after SCS [23].

In the mentioned North American trial by Markmann et al. NMP was performed directly after organ procurement with a portable device showing a significant reduction of ischemic bile duct complications whereas a comparable European trial by Nasralla et al. performed NMP after a relevant cold ischemic time at the recipient center without observing positive effects on bile duct complications in the further course [22, 24]. Accordingly, Mergental et al. showed that although the application of NMP in a “back-to-base” approach is able to rescue ECD organs, development of ischemic bile duct complications is not prevented, which is in line with the results of our study which showed significant damage of the bile ducts despite NMP after a prolonged cold ischemia time [25].

As NMP allows assessment of grafts under physiological conditions prior transplant, simultaneous evaluation of the degree of IRI and molecular mechanisms influencing short- and long-term cell damage should be a central issue of future experimental and clinical studies.

To our knowledge, this is the first investigation into the kinetics of pro- and anti-inflammatory mediators during NMP of porcine livers. Our results show an increase of IL-1α, IL-1β, IL-2, IL-6 and IL-18, with the latter being the only significantly elevated cytokine, during NMP after prolonged SCS. The statistically significant and early increase of IL-18 is particularly interesting as its role in IRI is perceived as indicator of caspase-1 activation, which is required for the release of both IL-18 and IL-1β from stressed cells. Takeuchi et al. showed a significant reduction of IRI and a concomitant upregulation of anti-inflammatory cytokines such as IL-4 and IL-10 in a mice model under blockade of IL-18 [26]. In line with these results, Bal et al. demonstrated a protective effect of IL-18-binding protein on IRI induced liver injury in an experimental rat model [27]. Of note, our data showed similar kinetics of IL-18 and GGT perfusate concentrations (early increase, peak after 2 hours and decrease during ongoing perfusion) after prolonged SCS suggesting an association between IL-18 secretion and bile duct inflammation or injury.

More importantly, HMGB1, one of the most widely analyzed DAMPs in the transplant setting, is released early during NMP and correlates with the conventionally measured cell damage of hepatocytes (i.e., AST and GLDH concentrations) and cholangiocytes (GGT concentration). Of note, the corresponding histological analyses did not corroborate our findings in terms of proof for significantly increased hepatic or biliary inflammation and injury, respectively, probably due to the short *ex-vivo* observational follow-up of only 6 hours in this model.

Ilmakunnas et al. introduced HMGB1 as an early marker of hepatic injury after transplantation, peaking as soon as 10 minutes after portalvenous reperfusion with the highest concentrations being observed in the caval effluent. HMGB1 kinetics did not correlate with either IL-6 or TNF-α, but with the degree of graft steatosis and postoperative ALT levels [28]. In addition, anti-HMGB1 antibodies were shown to be protective against IRI and subsequent hepatocellular damage and cytokine upregulation [29, 30].

However, the diagnostic value and mechanistic role of HMGB1 during NMP is still elusive. Scheuermann et al. showed that elevated levels of inflammatory molecules, such as HMGB1, are associated with increased activation of toll-like receptors and apoptosis after liver reperfusion in a rat model [31].

Interestingly, Scheuermann et al. and Goldaracena et al. described that the amount of recirculating inflammatory molecules increases with higher perfusate temperature during machine perfusion most likely as a result of increased cell metabolism [31, 32].

Of note, absence of filtration and/or adsorber systems in current NMP devices allows continuous perfusate recirculation and hence potential accumulation of metabolic products and inflammatory molecules.

Different strategies have been established to reduce the accumulation of pro-inflammatory molecules during machine perfusion. A simple but effective idea was published by Obara et al.: replacement of the initial perfusate after 5 minutes of subnormothermic machine perfusion as an attempt to mimic a filter or dialyzer led to significantly lower concentrations of transaminases and lactate levels after reperfusion in a porcine model [33]. Haemoadsorption with an incorporated cytokine filter has been used to reduce the inflammatory response during kidney machine perfusion resulting in improved renal blood flow, albeit without significantly influencing renal function [34]. With regard to lung machine perfusion, filter-based cytokine removal has been shown to decrease the development of pulmonary edema with uncertain effects on clinical pulmonary function post engraftment [35].

A critical issue of perfusate exchange and cytokine filters may be the simultaneous removal of not only pro- but also important anti-inflammatory mediators. Thus, specific antibodies might be more effective in order to improve grafts during perfusion. Garcia-Aroz et al. showed that livers treated with monoclonal antibodies against CD47 before perfusion following 30 or 60 min of warm ischemia time showed significantly lower ALT levels and higher bile production compared with their respective control groups [36]. Further potentially effective strategies might include the use of regulatory cytokines and cell therapies during NMP in order to create an anti-inflammatory environment for organ (re)conditioning.

Our study has some important limitations: Although our model reflects suboptimal storage conditions (SCS >12 h; commonly defined as marginal or ECD organs [1]), liver grafts from young and healthy pigs do not resemble conditions of (marginal/extended criteria) human donors.

Furthermore, to reduce costs and logistical complexity we applied whole blood in our perfusion protocol at the expense of limiting the comparability with the clinical setting. However, Liu et al. demonstrated a trend toward superior functional and hepatocellular injury outcomes, with even lower AST release for porcine liver NMP with whole blood when compared to red blood cells and steen solution [6].

In the era of machine perfusion, the monitoring of cytokine profiles and DAMPs during *ex-vivo* preservation and (re)conditioning of liver grafts might serve as useful biomarkers for detection of inflammation and relevant IRI. This would enable sophisticated analyses of specific therapeutic interventions in order to promote an anti-inflammatory environment and thereby reduce acute and chronic graft damage. Furthermore, the potential prognostic value of mentioned biomarkers for short- and long-term complications (such as biliary lesions with regard to the detrimental bile duct histology after prolonged SCS) could significantly improve organ assessment prior transplantation, despite the additional logistical and economic burden of corresponding analyses. Translational *ex-vivo* models, as presently described, but also long-term *in-vivo* models will play a crucial role in clarifying these aspects and optimizing machine perfusion based reconditioning protocols of marginal donor organs in the near future.

## Data Availability

The raw data supporting the conclusion of this article will be made available by the authors, without undue reservation.
